# Evaluation of calibration factor of OSLD toward eye lens exposure dose measurement of medical staff during IVR

**DOI:** 10.1002/acm2.13042

**Published:** 2020-10-14

**Authors:** Takashi Asahara, Hiroaki Hayashi, Sota Goto, Natsumi Kimoto, Kazuki Takegami, Tatsuya Maeda, Yuki Kanazawa, Tohru Okazaki, Takuya Hashizume

**Affiliations:** ^1^ Division of Health Sciences Graduate School of Medical Sciences Kanazawa University Kanazawa Japan; ^2^ Division of Radiology Medical Support Department Okayama University Hospital Okayama Japan; ^3^ College of Medical, Pharmaceutical and Health Sciences Kanazawa University Kanazawa Japan; ^4^ Graduate School of Biomedical Sciences Tokushima University Tokushima Japan; ^5^ Nagase Landauer, Ltd. Tsukuba Japan

**Keywords:** calibration factor, eye lens dose, IVR, OSL dosimeter, scattered x‐ray

## Abstract

The eye lens is a sensitive organ of which an x‐ray exposure dose should be managed during interventional radiology (IVR). In the actual situations, the eye lens is exposed to scattered x‐rays; they have different from the standard x‐ray energies which are used for general dose calibration of the dosimeter. To perform precise dose measurement, the energy dependence of the dosimeter should be properly accounted for when calibrating the dosimeter. The vendor supplies a calibration factor using 80‐kV diagnostic x‐rays under a free‐air condition. However, whether it is possible to use this calibration factor to evaluate the air kerma during the evaluation of the eye lens dose is unclear. In this paper, we aim to precisely determine calibration factors, and also examine the possible application of using a vendor‐supplied calibration factor. First, the x‐ray spectrum at the eye lens position during fluoroscopy was measured with a CdTe x‐ray spectrometer. We mimicked transfemoral cardiac catheterization using a human‐type phantom. Second, we evaluated the doses and calibration factors at three dosimetric points: front and back of protective goggles, and the front of the head (eye lens position). We used the measured x‐ray spectrum to determine the incident photon distribution in the eye lens regions, and x‐ray spectra corresponding to the dosimetric points around the eye lens were estimated using Monte Carlo simulation. Although the calibration factors varied with dosimetric positions, we found that the factors obtained were similar to the vendor‐supplied calibration factor. Furthermore, based on the experiment, we propose a practical way to calibrate an OSL dosimeter in an actual clinical situation. A person evaluating doses can use a vendor‐supplied calibration factor without any corrections for energy dependences, only when they add a systematic uncertainty of 5%. This evidence will strongly support actual exposure dose measurement during a clinical study.

## 1 INTRODUCTION

Currently, x‐ray examinations are an essential technology for performing noninvasive medical diagnosis. During procedures, such as interventional radiology (IVR), medical staff are routinely exposed to scattered x‐rays,^1^, ^2^, ^3^ and many dose evaluators warn that medical doctors performing IVR procedures receive a large amount of x‐ray exposure. For these doctors, reducing radiation‐induced cataracts^4^, ^5^, ^6^ should be a top priority, and thus in 2011, the International Commission on Radiation Protection (ICRP) recommended a new radiation dose limit of 20 mSv per year.^7^, ^8^ Based on this new recommendation, research concerning eye lens dosimetry has been very active.^9^, ^10^, ^11^ In these studies, the eye lens doses were often evaluated based on indirect measurements using a personal dosimeter. Generally, a personal dosimeter can determine radiation type and energy by means of a special algorithm based on the differences of responses related to different radiation filters. Personal dosimeters focus on effective dose evaluation of the whole body, however, this is not suitable for directly evaluating the dose to a specific organ, such as the eye lens. In contrast, we focused our attention on the direct dose measurement of the eye lens precisely under actual conditions.

In order to achieve a direct dose measurement of the eye lens, a small‐type OSL dosimeter (nanoDot^TM^, 10 mm × 10 mm × 2 mm^t^) ^12^ is available. Although small‐type OSL dosimeters do not have the ability to estimate both radiation type and energy, a person evaluating doses does not need information concerning radiation type because during IVR procedures medical staff are only exposed to x‐ray photons. Thus, we need to only pay attention to the energy dependence^13^, ^14^ of the small‐type OSL dosimeter. Basically speaking, the energy dependence of the dosimeter is the difference of mass energy‐absorption coefficients between the OSL dosimeter and air^15^; this means that energy dependence for polychromatic x‐ray distribution should be analyzed with polychromatic x‐rays and should not be treated as monochromatic x‐rays.^16^

The dose to the eye lens is mainly caused by scattered x‐rays from the patient.^3^, ^17^ This means that a person evaluating doses should pay close attention to the verifications of the polychromatic x‐ray spectrum related to the scattered and penetrating x‐rays. Moreover, for eye lens dose evaluation, the following special attention should be paid. During the current clinical diagnosis, medical doctors usually wear the protective goggles to reduce eye lens exposure. When eye lens dose is evaluated by a dosimeter, a person evaluating doses should consider the change in x‐ray energy caused by the beam hardening effect^18^ during the penetration of materials (goggles). Furthermore, effect of contamination from backscattering x‐rays generated by the head of the operator has to be considered. There are no previous studies in which these phenomena are taken into consideration for calibrating a dosimeter used to measure exposure dose to the eye lens.

Recently, we have proposed a precise calibration procedure in which we considered the polychromatic x‐ray distribution and the energy dependence of an OSL dosimeter.^16^ In this study, we applied this general method to a specific situation of eye lens dosimetry, and aimed to evaluate the precisely determined calibration factors. Because many clinical studies use a vendor‐supplied calibration factor, we also investigated the applicability of this factor. The results derived from this study will play an important role in determining the usefulness of clinical data.

## 2 MATERIALS AND METHODS

### 2.A Calibration procedure taking into consideration energy dependence of the OSL dosimeter

We will explain a procedure to determine dose calibration factors by taking into consideration the energy dependence of an OSL dosimeter and differences found in various x‐ray spectra. Our previous study^16^ aimed to derive a calibration factor used for general x‐ray diagnosis, and the effects of beam hardening, scattered x‐rays, and backscattering x‐rays were evaluated. Namely, when an x‐ray spectrum including the above effects is obtained, a proper calibration factor can be determined. In this study, we applied this procedure to eye lens dosimetry.

The following is a brief explanation of our calibration method. The calibration factor of the OSL dosimeter is defined as the ratio of air kerma divided by the absorbed dose corresponding to the dosimeter. Then, the “calculated calibration factor (CCF)” can be determined using the following formula:

(1) $$\text{Calculated calibration factor}:\text{CCF}={\left(\frac{\text{Air kerma}}{\text{Absorbed dose}}\right)}_{\text{spectrum}}.$$

From a unique x‐ray spectrum, both air kerma and absorbed dose can be calculated simultaneously. Then, air kerma and absorbed dose are calculated as follows:

(2) $$\text{Air kerma}=\int C\left(E\right)\times E\times \frac{\mu_{\text{en}}\left(E\right)}{\rho }\text{dE}=\int K\left(E\right)\text{dE},$$

(3) $$\text{Absorbed dose}=\int K\left(E\right)\times \text{Eff}.\left(E\right)\text{dE}=\int D\left(E\right)\text{dE},$$

where C(E), E, and μ_en_(E)/ρ are the intensity of x‐ray spectrum, energy, and mass energy‐absorption coefficient of air, respectively. Eff.(E) is the energy dependence of the OSL dosimeter.^13^ It is important that we determine a calibration factor when deriving the x‐ray spectrum. The calibration factor described in Eq. (1) can provide an absolute value, but in a practical case, only a measurable value (the response of the OSL dosimeter: counts), which is proportional to the absorbed dose, can be obtained. In actual analysis when using an OSL dosimeter reading device, relative responses can be read out. In order to analyze the relationship between an absolute value and a relative value, we need to perform additional experiments to derive an actual calibration factor. Here, the vendor‐supplied calibration factor used for a commercially available OSL dosimeter (nanoDot^TM^, Landauer, Inc., Illinois, USA) was determined using 80‐kV diagnostic x‐rays with a half‐value layer (HVL) of 3.01 mm aluminum; this quality of x‐ray is known as an RQR6 beam.^19^ Using this radiation quality, the vendor provides the calibration factors for air kerma, shallow dose equivalent (SDE: H_P_(0.07)), and deep dose equivalent (DDE: H_p_(10)). In this study, we focused our attention on a method for determining the calibration factor measured by air kerma. In a similar way, we determined the conversion coefficient *f* at 80 kV x‐rays as follows:

(4) $${\left(\frac{\text{Air kerma}}{\text{Counts}/\varepsilon }\right)}_{\exp.\left(80\;\text{kV}\right)}=f\times {\left(\frac{\text{Air kerma}}{\text{Absorbed dose}}\right)}_{\text{spectrum}\left(80\;\text{kV}\right)}=f\times \text{CCF,}$$

where “Counts” and “ε” are the response of the OSL dosimeter and intrinsic detection efficiency for each dosimeter, respectively. An experiment for the determination of the conversion coefficient *f* will be described later.

### 2.B Estimation of x‐ray spectra at dosimetric points

**Fig. 1:**
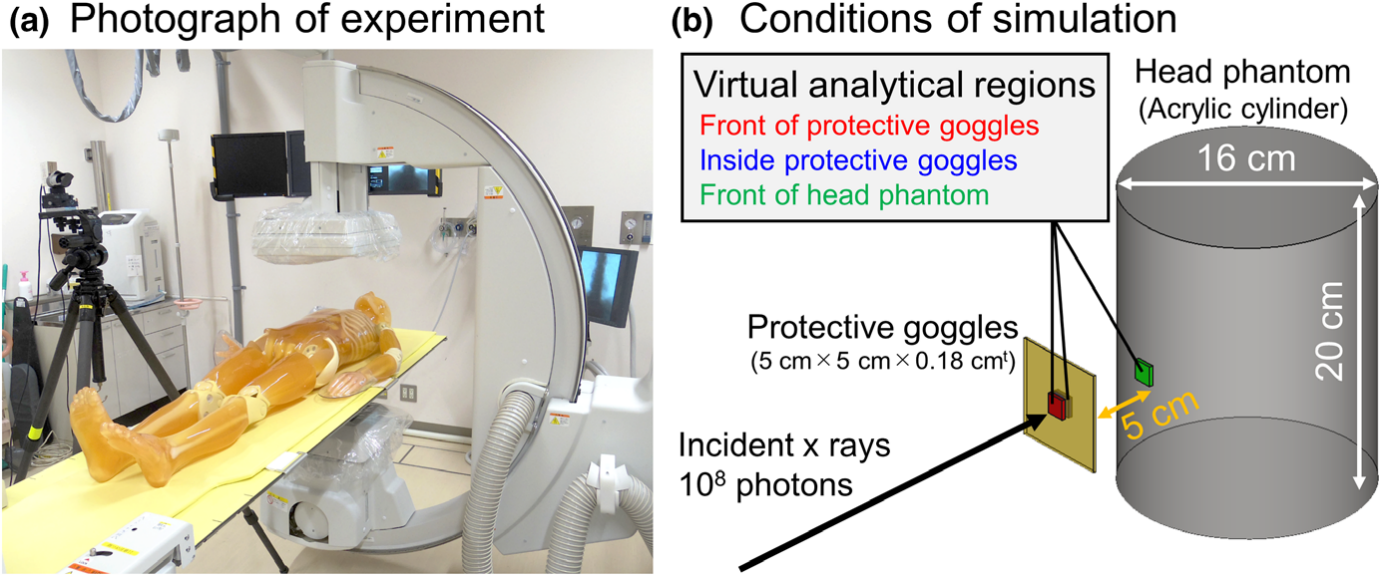
(a) photograph of experimental arrangement to measure x‐ray spectrum at eye lens position during fluoroscopic examination. (b) condition of simulation to calculate x‐ray spectra of the virtual analytical regions at the front and inside of the protective goggles, and front of the head phantom.

We will describe an experiment and a simulation used to obtain the x‐ray spectra around the eye lens position. First, we performed a phantom experiment as shown in Fig. 1(a); clinical application of an IVR procedure was mimicked using a human body equivalent phantom (PBU‐60, Kyoto Kagaku Co., Ltd., Kyoto, Japan) as a patient. The x‐ray source for the fluoroscopic equipment (versiFlex VISTA, Hitachi, Ltd., Tokyo, Japan), which was an under the table source type, was used. The distance between the focal spot and the center of the human body equivalent phantom was set at 40 cm. The followings are irradiation conditions: tube voltage of 74 kV (tungsten target with total filtration of 2.5 mm aluminum), tube current of 0.9 mA, exposure time of 30 min, and a frame rate of 15 frames per second (fps). An automatic exposure control system and automatic settings for determination of tube voltage installed on this equipment were used. The irradiation field was set to the cardiac area, and the field size was 30 cm × 25 cm. In order to measure the x‐ray spectrum, we used a CdTe spectrometer (123‐0 type radiation detector, EMF Japan Co., Ltd., Hyogo, Japan) which was placed at the eye lens position of a 170‐cm‐tall human dummy. The Compton scattering spectroscopic procedure proposed by Maeda et al.^20^ was applied, and taking into account the response function of a CdTe detector and using the Klein–Nishina formula, necessary corrections to obtain x‐ray spectrum were appropriately applied.

**Fig. 2:**
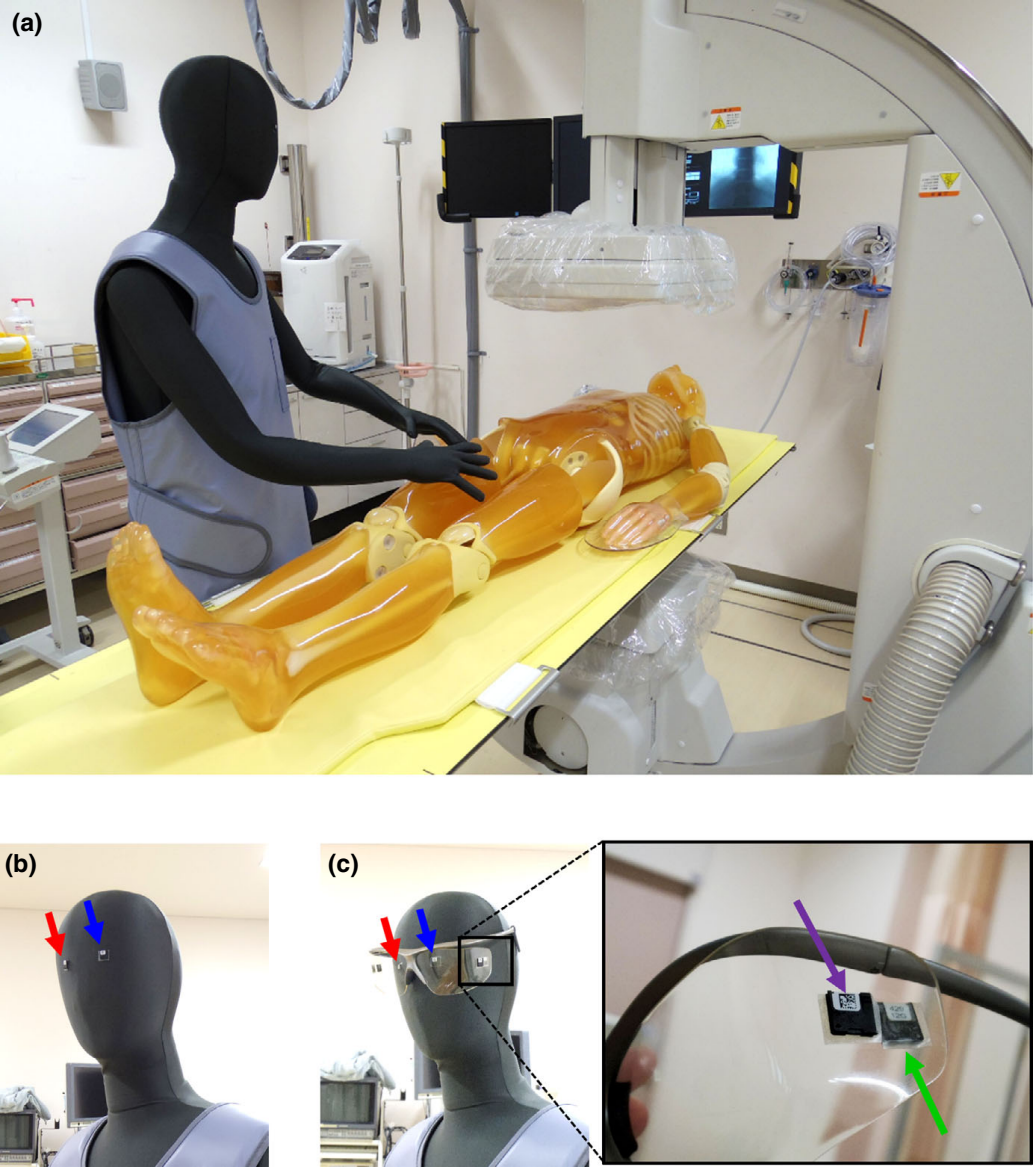
Photographs of demonstration for measuring eye lens dose during fluoroscopic examination. (a) photograph of experimental arrangement to mimic transfemoral cardiac catheterization procedure using a human dummy and a human equivalent phantom. We performed two experiments: without protective goggles represented in (b) and with protective goggles represented in (c).

In order to estimate the effects of x‐ray attenuation in protective goggles and the effect of contamination of backscattering x‐rays from the head of the operator, a Monte Carlo simulation was performed using EGS5 (electron‐gamma‐shower version 5) code.^21^ Figure 1(b) shows a schematic drawing of the simulation conditions. Instead of an actual human head, a cylindrical acrylic phantom was constructed: the height being 20 cm, diameter $16\;{\text{cm}}^{\phi }$, and density 1.000 g/cm^3^. An absorber (atomic components of acrylic and lead oxide are 72.5% and 27.5% with density of 2.0 g/cm^3^) which is made of the same materials as protective goggles having a thickness of 0.5 mm Pb equivalent material^22^ was placed in front of the head phantom. The distance between the protective goggles and the head phantom was set at 5 cm, which was determined from the phantom experiment when the human dummy puts on the goggles as described later [see Fig. 2(c)]. Three virtual analytical regions for evaluating the incident x‐ray spectra were set: (1) front of the protective goggles, (2) inside of the protective goggles, and (3) front of the head phantom (eye lens position). The x‐ray spectrum measured with a CdTe spectrometer [see Fig. 1(a)] was used to determine photon distribution of the generated x‐rays. The total number of incident x‐rays was 10^8^, and the irradiation field was set at 16 cm × 16 cm. In the simulation, the incident x‐ray spectrum for each of the virtual analytical regions was recorded.

### 2.C Experiment to determine the actual calibration factor

In this section, we will describe the experimental procedure to determine the conversion coefficient *f* in Eq. (4). Because the conversion coefficient *f* is only effected by the counting efficiency of the reading device, it can be determined from the experiment, in which x‐ray exposure to the OSL dosimeter was performed using diagnostic x‐ray equipment.

The experimental value of Counts/ε and air kerma was obtained using an OSL dosimeter and an ionization chamber, respectively. Diagnostic x‐ray equipment (MRAD‐A 50S/70, Canon Medical Systems Corp., Tochigi, Japan) was used. In order to reduce contamination from scattered x‐rays generated from the movable diaphragm of the x‐ray equipment, the OSL dosimeter and ionization chamber were individually placed in a lead shielding box having a window opening of $100\;{\text{mm}}^{\phi }$ and an additional lead collimator ($25\;{\text{mm}}^{\phi }$) was set in front of the x‐ray equipment.^23^ The distance between the x‐ray focal point and the dosimeter was set at 200 cm. Irradiation conditions were 80 kV (tungsten target with total filtration of 2.5 mm aluminum), 100 mA, and 2 s. The response of the OSL dosimeter was defined as Counts/ε; where “Counts” and “ε” were measured response and intrinsic detection sensitivity, respectively.^13^, ^16^ To reduce statistical uncertainty, three OSL dosimeters were individually irradiated at the above conditions, and each dosimeter was read five times using a commercially available reading device (microStar, Landauer, Inc., Illinois, USA). We adopted the mean value of Counts/ε for 15 readings. The signal loss caused by multiple readings was corrected.^12^, ^24^ Here, we used an “unscreened”‐type nanoDot OSL dosimeter, and we determined the accuracy related to the sensitivity of each nanoDot OSL dosimeter. According to previous research,^25^ the accuracy when using an unscreened‐type nanoDot OSL dosimeter was evaluated to be 5.5%.

The air kerma corresponding to the same irradiation conditions was measured with an ionization chamber (DC‐300, PTW, Freiburg, Germany). To calculate air kerma from the measured electric charge, the following values were used: a calibration constant of 2.84 × 10^5^ [(C/kg)/C] for 80 kV x‐rays and a W/e of 33.97 [J/C].^26^ The corrections for room temperature and air pressure during the measurement were performed so as to match a standard temperature of 295 K and a pressure of 1013 hPa.^27^ The air kerma was measured five times, and the mean value of air kerma was adopted. The mean value of Counts/ε was approximately 14,500 counts, and the averaged value of air kerma was determined to be approximately 3 mGy. Using the response of the OSL dosimeter and air kerma, the conversion coefficient *f* in Eq. (4) was calculated. Then, the correlation between the calibration factor and effective energy of x‐ray spectra was examined. When the x‐ray spectrum is obtained, the corresponding air kerma can be calculated. Additionally, the calculation procedure for attenuation related to aluminum is well known. Combining this knowledge, the air kerma corresponding to the x‐ray spectrum after penetrating the aluminum having thickness t can be described as follows:

(5) $$\text{Air kerma}\left(t\right)=\int C\left(E\right)\times E\times {\left(\frac{\mu_{\text{en}}\left(E\right)}{\rho }\right)}_{\text{air}}\times e^{‐\mu {\left(E\right)}_{\text{Al}}\times t}\text{dE},$$

where C(E), ${\left(\mu_{\text{en}}\left(E\right)/\rho \right)}_{\text{air}}$ and μ(E)_Al_ are the intensity of measured x‐ray spectrum, the mass energy‐absorption coefficient of air and the linear attenuation coefficient of aluminum, respectively.^28^ In order to obtain the effective energy, a virtual experiment was performed and the half‐value layer (HVL) was obtained from the attenuation curve using air kerma. Here, since the HVL is the amount of attenuation corresponding to the effective energy of the continuous x‐ray spectrum, the effective linear attenuation coefficient μ_eff_ can be derived from the following relationship:

(6) $$\mu_{\text{eff}}=\frac{\ln\left(2\right)}{\text{HVL}}.$$

Then, because μ has a unique relationship with energy E for monoenergetic x‐ray,^28^ μ_eff_ obtained from the above equation can be converted to effective energy using this relationship.

### 2.D Demonstration of eye lens dosimetry during fluoroscopic examination

In order to demonstrate the applicability of our calibration procedure, we performed a phantom study using an OSL dosimeter. Figure 2 shows photographs of the experiment. Figure 2(a) shows experimental arrangement; an IVR procedure of transfemoral cardiac catheterization was mimicked. A clinical fluoroscopic system (versiFlex VISTA, Hitachi, Ltd., Japan) was used, and a human body phantom (PBU‐60, Kyoto Kagaku Co., Ltd., Kyoto, Japan) was placed on a fluoroscopic table. Instead of an actual operator, we used a human dummy consisting of expanded polystyrene. We should note that backscattering x‐rays are not generated from the human dummy and the component of backscattering x‐rays is corrected for by using simulation data. The irradiation field was set to the cardiac position. The experimental conditions were the same as described in Section 2.B. Based on an automatic exposure control system, the following parameters were adopted: 74 kV, 0.9 mA, 30 min irradiation, and 15 fps. In order to demonstrate the usefulness of wearing protective goggles, we performed two experiments; (1) the operator does not use protective goggles as shown in Fig. 2(b), and (2) the operator uses protective goggles with a 0.5 mm Pb equivalent material (Panorama Shield Extrawide, Toray Medical Co., Ltd., Tokyo, Japan) as presented in Fig. 2(c). The OSL dosimeters were attached to the right and left eye lens positions. The red and blue arrows in Figs. 2(b) and 2(c) correspond to the dosimetric positions of right and left eye lenses, respectively. For the experiment using protective goggles, we attached additional OSL dosimeters to the following dosimetric positions; front and back of the goggles. These OSL dosimeters were attached on the outside and inside of the protective goggles as shown with purple and green arrows, respectively, in Fig. 2(c). Because in actual clinical dosimetry, dosimeters cannot be placed on the surface of the eye, the positions of front and back of the goggles are considered to be applicable in the actual clinical studies. The measured responses of the OSL dosimeters were analyzed using precise calibration factors determined by our method.

**Fig. 3:**
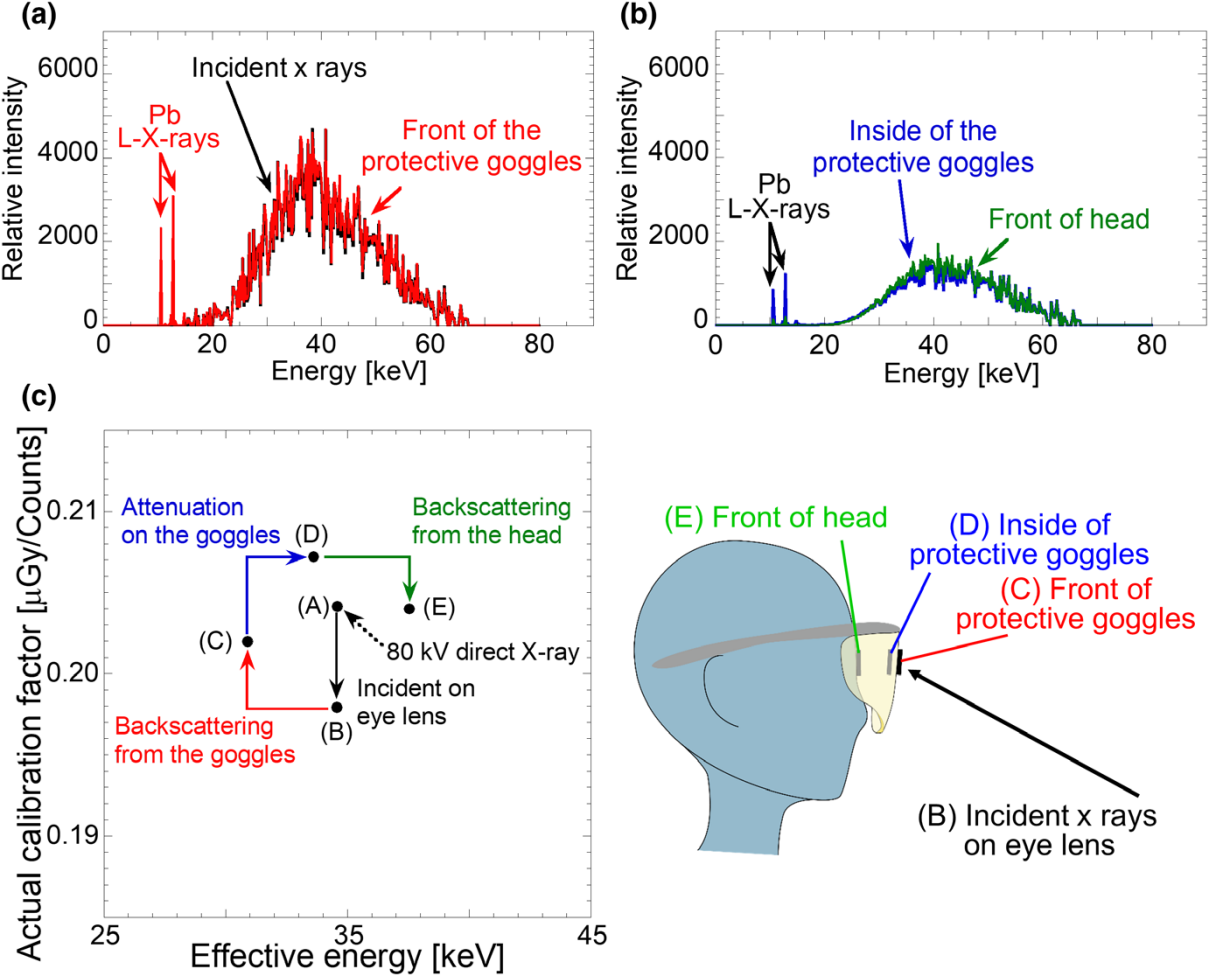
X‐ray spectra and calibration factors around the eye lens position. (a) and (b) are x‐ray spectra before and after penetrating protective goggles, respectively. (c) is the relationship between effective energy and calibration factor for each dosimetric position. The calibration factors were determined using x‐ray spectra represented in (a) and (b).

## 3 RESULTS

### 3.A X‐ray spectra and dose calibration factors around the eye lens position

Figure 3 shows the x‐ray spectra results and actual calibration factors for each dosimetric point shown in the inset. Figures 3(a) and 3(b) correspond to the x‐ray spectra before and after penetrating protective goggles, respectively. The black line shows the x‐ray spectrum being incident to the eye lens position; this spectrum was experimentally acquired with the phantom experiment presented in Fig. 1(a). For this spectrum, the correction for the response function of the CdTe spectrometer and application of the Klein–Nishina formula were performed. The red, blue, and green lines represent x‐ray spectra corresponding to the dosimetric positions of front and inside of the protective goggles, as well as the front of head (eye lens position), respectively. The corresponding x‐ray spectra were obtained by Monte Carlo simulation in which the experimentally measured spectrum (black line) is imported as incident x‐ray distribution. It is an interesting point that characteristic L‐x‐rays, caused by the interaction between incident x‐rays and lead within the protective goggles, were clearly observed in the x‐ray spectra at the dosimetric positions around the protective goggles. Although the tube voltage of the fluoroscopic equipment was set at 74 kV, the observed maximum energy was measured as 65 keV. This can be explained by variations in x‐ray energy caused by the Compton scattering effect.

The conversion coefficient *f* in Eq. (4) was determined to be 6.551 × 10^−4^. Using this conversion coefficient *f*, the actual calibration factors were derived from the calculated calibration factor (CCF) that is defined in Eq. (1). Figure 3(c) shows the relationship between actual calibration factors and effective energies. The vendor‐supplied calibration factor value using 80 kV x‐ray was plotted at the center (A) of the graph. Plot point (B) shows the calibration factor of incident x‐rays which were scattered by the patient, and was 3% smaller than the vendor‐supplied calibration factor. It was reported that the energy of scattered x‐rays was changed by <10 keV from that found in the previous research.^17^, ^29^ In the specific case of this study, the effective energy of the scattered x‐ray spectrum being incident to the eye lens [related to point (B)] is similar to that of 80 kV [related to point (A)]. Furthermore, it is very interesting that the calibration factors show different values even though they have similar effective energies. When the dosimeter was attached to the outside of the protective goggles, contamination from the backscattering x‐rays from the goggles should be considered. We found that the contribution of the L‐x‐rays are not negligible. In this case, the calibration factor and the effective energy are also varied as shown in plot point (C). The main component of backscattering x‐rays from goggles is the L‐x‐rays of lead. For the x‐rays after penetrating the protective goggles, the calibration factor and the effective energy become larger as shown in plot point (D); this is due to the beam hardening effect. Finally, when the backscattering x‐rays from the head of the operator were considered, the calibration factor became lower as presented in plot point (E). In Fig. 3(c), we found that the calibration factors used for eye lens dosimetry plotted close to the vendor‐supplied factor, and all of the calibration factors are within 5% of the vendor‐supplied factor. From these results, we recommend the acceptance of a systematic uncertainty of 5% when a person evaluating doses uses vendor‐supplied calibration factors for eye lens dosimetry during an IVR procedure.

### 3.B Results of demonstration for eye lens dosimetry during fluoroscopic examination

**Fig. 4:**
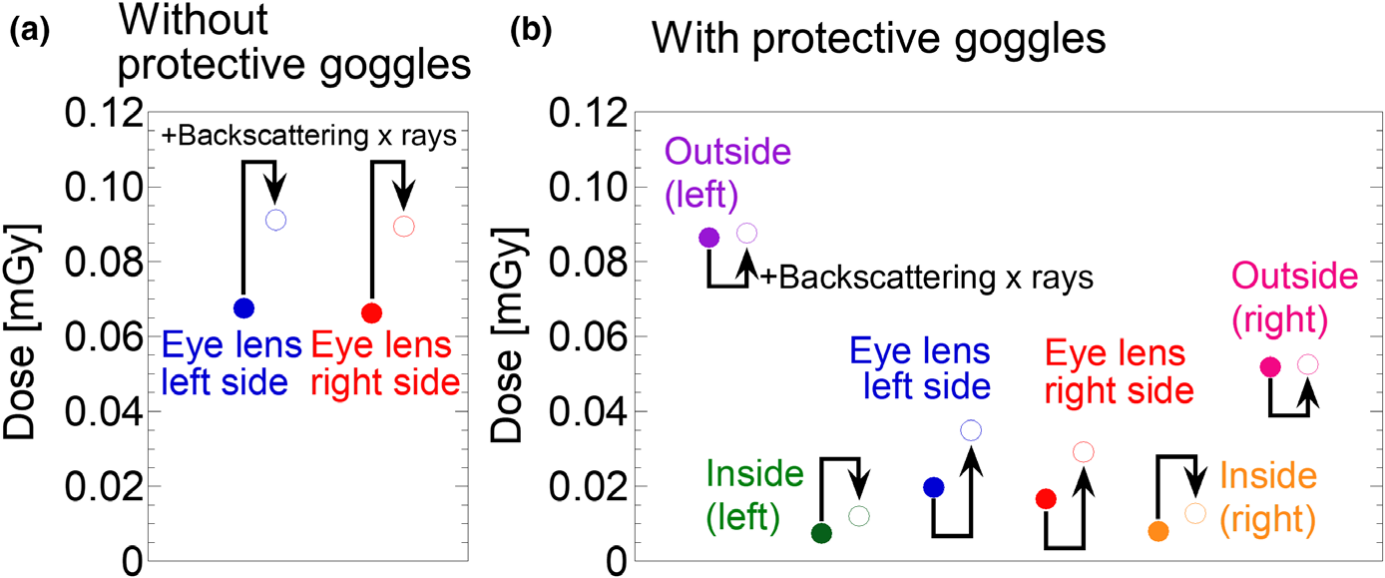
Measured dose around eye lens position during fluoroscopic examination. (a) and (b) correspond to the eye lens doses without and with protective goggles, respectively. The closed circles are the dose measured in our phantom experiment, and the open circles are the dose estimated the effect of backscattering x‐rays from the head. The doses were analyzed based on a precisely determined calibration factor (our method). The benefits of using protective goggles can be clearly observed.

Here, we will demonstrate the eye lens dosimetry based on precisely determined calibration factors. Figure 4 shows the results of the experiment for eye lens dosimetry during fluoroscopic examination. We measured the eye lens dose using small‐type OSL dosimeters. The experimental arrangement is shown in Fig. 2, and the radiation dose was analyzed using our method. In our experiment, a human dummy consisting of expanded polystyrene was used instead of an actual operator, therefore the effect of backscattering x‐rays from the human head on the measured dose should be additionally estimated. In order to estimate the effect of the backscattering x‐rays, we analyzed the contribution of the effect using Monte Carlo simulation. Table 1 shows a summary of the contribution of the backscattering x‐rays determined by the simulation. A correction factor was derived from the ratio of the air kerma at each dosimetric position with and without the head phantom. The closed circles are the doses analyzed from the phantom experiment, and the open circles are dose correction data for backscattering x‐rays. Figure 4(a) shows the results of eye lens dose when the operator does not use protective goggles; blue and red circles correspond to the eye lens doses of left and right sides, respectively. As shown in Fig. 4(b), the doses around the left and right eye lens positions are drastically reduced when the operator uses protective goggles. The purple and green circles represent the doses outside and inside the protective goggles on the left side, respectively. The pink and orange circles correspond to the doses outside and inside the goggles on the right side, respectively. Our result demonstrates the benefits of using protective goggles. When protective goggles are used, the eye lens dose was reduced by approximately 30% for both left and right sides.

**Table 1:** Summary of correction factor of backscattering x‐rays.

Condition	Position	Correction factor
Without protective goggles	Eye lens	1.35
With protective goggles	Eye lens	1.77
	Outside of protective goggles	1.01
	Inside of protective goggles	1.62

## 4 DISCUSSION

In this paper, we determined a calibration factor for a small‐type OSL dosimeter in order to achieve a precise evaluation of the eye lens dose during fluoroscopic examination. By considering the variation of x‐ray spectra under actual conditions and the energy dependence of the OSL dosimeter, the calibration factors of outside and inside protective goggles, and the eye lens position were determined. We found it is very meaningful to determine the calibration factors precisely for each place. We also found the usefulness of the calibration factor provided by the vendor. We will discuss the details below.

### 4.A Usefulness of vendor‐supplied calibration factor for eye lens dosimetry

We will discuss the calibration factors of the OSL dosimeter determined in this study. We found it interesting that the calibration factors concerning eye lens dosimetry were similar to the vendor‐supplied calibration factor as shown in Fig. 3(c). When the calibration factors of incident x‐rays at the eye lens [related to point (B)] and standard x‐rays of 80 kV [related to point (A)] were compared, it can be seen that evaluation using effective energy is not sufficient. A calibration factor needs to be determined taking into consideration both the x‐ray spectrum and the sensitivity of the OSL dosimeter. Although the factors varied in range of 0.198–0.208 as shown in Fig. 3(c) and these calibration factors include the effects of energy variation, contamination of scattered x‐rays, beam hardening effect, etc.; these various effects seem to balance out coincidentally. Recently Tanaka et al. reported the measured dose of scattered x‐rays during IVR procedure, and in their study dose calibration was performed using 80 kV x‐rays.^30^ In our paper, we verified that the calibration factor determined using standard x‐rays (80‐kV direct x‐rays) can be applied to measure the dose during IVR, therefore our result justifies the previously measured data. Here, the standard calibration factor in this study was derived using 80 kV x‐rays (effective energy of 32.2 keV). When the person evaluating a dose wants to use the calibration factor determined by the RQR6 beam (effective energy of 33.5 keV), it is noted that there is a difference between the calibration factors for the RQR6 beam (0.202) and our experimental condition (0.204) therefore consideration is needed. If a person who needs to evaluate the exposure doses precisely, the factors at dosimetric positions (B), (C), (D), and (E) should be applied to the analysis. This analysis can be established under the following limitation; these calibration factors can only be applied to static conditions, which are similar to our experimental conditions. The relationship between the x‐ray source and the dosimetric point becomes dynamic for actual clinical dosimetric conditions because of movement by the medical staff. Thus, it is rare that the conditions used in our experiment agree exactly with actual clinical results.

It is known that OSL dosimeters have relatively large energy dependence in the lower energy region,^13^, ^14^, ^15^ and it is considered that calibration factors used for eye lens dosimetry during IVR procedures should be determined as described above. However, not all dose evaluators can use the above‐mentioned procedure in which the energy dependence of the OSL dosimeter is carefully considered. Here, we propose a practical procedure based on our results as follows. The results in Fig. 3 indicate that the difference between a vendor‐supplied calibration factor and the factors determined in this study are within 5%. This means that the vendor‐supplied calibration factor can be applied to the dose evaluation of eye lens during an IVR procedure under the following limitation; a person evaluating doses should add a systematic uncertainty of 5%. This is a valuable conclusion that was derived based on the research explained in this paper. In our experiment, the tube voltage of 74 kV determined by the automatic exposure control system was used. When different x‐ray qualities are used, the energy dependence of the OSL dosimeter needs to be considered. As mentioned above, because the energy dependence of the OSL dosimeter is large,^13^, ^14^, ^15^ we need to pay special attention to this phenomenon.

In this study, we focused our attention on energy dependence as it affects the calibration factor, and we did not consider the effect of angular dependence. Even if x‐rays are incident on the dosimeter obliquely, there is almost no change in the sensitivity, but when x‐rays enter the dosimeter from the lateral direction, a very large decrease in sensitivity is observed. This effect was known as “angular dependence”.^25^, ^31^, ^32^ In clinical cases, it is difficult to identify the incident angle of x‐rays to the dosimeter, therefore the effect of angular dependence becomes one of the uncertainties. Additional special attention is needed for the consideration of energy dependence when there is the possibility that x‐rays are incident to the side of the dosimeter. In our static experiment, the condition that x‐rays were incident from the side of the dosimeter was not used, so the results could be calculated without considering angular dependence. When we measure doses under clinical conditions, the effect of angular dependence, especially the possibility of x‐ray incidence from the side direction, should be taken into consideration.

### 4.B Evaluation of the measured dose around the eye lens position

We will discuss the eye lens doses measured using an OSL dosimeter in the phantom experiment. Using precise calibration factors determined in Fig. 3(c), we evaluated the effect of protective goggles on dose reduction. As shown in Fig. 4, the presence of protective goggles was clearly observed. In our experiment, the reduction rate of exposure dose using protective goggles was analyzed to be 70%. However, basically speaking, the reduction rate using the protective goggles depends on the design of the goggles and analytical procedure.^33^, ^34^ Even though a 70% reduction was one of the experimental results, we recommend wearing goggles when performing IVR procedure. In actual situations, the radiation dosimeter may not be attached directly to an actual eye lens. In this case, the eye lens dose may be inferred from the data at other dosimetric positions; for example, the outside and inside positions of the protective goggles are candidates. This is a limitation of this evaluation. In our experiment, we used a human dummy in order to imitate actual body positions during an IVR procedure. Contribution of backscattering x‐rays was not included because the human dummy does not consist of human equivalent material. Based on our simulation result, the contribution of backscattering x‐rays is estimated to be 35% at eye lens position and that corrected data are presented in Fig. 4. We expect that accurate clinical data will be obtained with precise calibration using our data.

The ICRP recommended to use operational quantity for monitoring eye lens dose using a dose equivalent at 3 mm depth: H_p_(3)^35^, ^36^ instead of air kerma. We will describe the difference between air kerma and H_p_(3). The H_p_(3) is calculated using the following formula:

(7) $$H_{p}\left(3\right)=\text{Air kerma}\times h_{\text{pK}}\left(3\right),$$

where h_pK_(3) is the conversion coefficient from air kerma to H_p_(3). The h_pK_(3) depends on the energy and the angle of incident x‐rays. In addition, the size and shape of the calibration phantoms are important factors in the determination of h_pK_(3).^37^ There have been many studies on these investigations. International standards of IEC 62387‐2012, ISO 4037‐1, and 12789‐2 referred the conversion coefficient at 3‐mm depth using a slab phantom made of acrylic or soft‐tissue equivalent material for calibration.^38^, ^39^, ^40^ Moreover, the shape of the phantom has been reported elsewhere.^29^, ^37^, ^41^ In this paper, we reported a precise calibration procedure of measuring “air kerma.” In order to evaluate eye lens dose, air kerma should be converted to the H_p_(3). On the other hand, originally measured air kerma is valuable for radiation protection research. Here, although the accuracy of the dose estimation at eye lens is lower, it is noted that H_p_(0.07) and H_p_(10) are also useful for estimating H_p_(3) when the radiation measurement fields are known in advance.^42^ For example, when scientists want to evaluate the efficiency of dose reduction using currently improved radiation protection techniques, the evaluation based on air kerma is useful because the value directly reflects the absorbed dose at measured points. In order to perform that study, not only eye lens dose in terms of H_p_(3) but also data for other dosimetric positions in terms of air kerma should be measured. We expected that the evidence and procedure play an important role for obtaining accurate value using OSL dosimeter.

## 5 CONCLUSIONS

In order to achieve precise dose evaluation of the eye lens of an operator when using an OSL dosimeter for the actual clinical measurement, we determined calibration factors. Our method took into consideration the difference in x‐ray spectra and the energy dependence of the dosimeter. Calibration factors at three dosimetric positions were evaluated: front and inside of the protective goggles, and eye lens. We found that the values of calibration factors varied for vendor‐supplied factor which was determined using 80 kV x‐rays. In the case that a person evaluating doses can apply our results, the exposure doses can be determined precisely. On the other hand, when they cannot apply our results because of restrictions related to actual clinical situations, we proposed a practical way. A person evaluating doses can use the vendor‐supplied calibration factor with a systematic uncertainty of 5%. Note that this conclusion can be applied to a fluoroscopic system generating 74 kV x‐rays (determined by an automatic exposure control system). Strictly speaking, when we want to evaluate doses using other x‐ray equipment in which different x‐ray qualities are applied, energy dependence should be accounted for.

## CONFLICT OF INTEREST

The authors of T. Okazaki and T. Hashizume are employees of Nagase Landauer, Ltd., Japan.

## AUTHORS' CONTRIBUTIONS

T. Asahara carried out this research mainly and contributed greatly to the writing of the scientific paper. H. Hayashi summarized this research as a manager. S. Goto conducted a dosimeter experiment. N. Kimoto analyzed data and helped to write a paper. K. Takegami helped to perform experiments using OSL dosimeters. T. Maeda assisted in data analysis. Y. Kanazawa provided guidance in research execution and paper writing. T. Okazaki conducted a detailed information survey on the x‐ray field. T. Hashizume performed basic research on eye lens protective goggles.
